# Punicalin Attenuates Breast Cancer-Associated Osteolysis by Inhibiting the NF-κB Signaling Pathway of Osteoclasts

**DOI:** 10.3389/fphar.2021.789552

**Published:** 2021-11-12

**Authors:** Tao Li, Guangyao Jiang, Xuantao Hu, Daishui Yang, Tingting Tan, Zhi Gao, Zhuoyuan Chen, Cheng Xiang, Shizhen Li, Zhengxiao Ouyang, Xiaoning Guo

**Affiliations:** ^1^ Department of Orthopedics, The Second Xiangya Hospital, Central South University, Changsha, China; ^2^ Department of Immunology, Xiangya School of Medicine, Central South University, Changsha, China; ^3^ Department of Geriatrics, The Second Xiangya Hospital, Central South University, Changsha, China

**Keywords:** punicalin, osteoclast, osteoporosis, osteolysis, NF-κB, breast cancer, bone metastasis

## Abstract

**Background:** Breast cancer bone metastasis and osteoporosis are both severe diseases that seriously threaten human health. These diseases are closely associated with osteolytic lesions. And osteoclasts are the key targets of this pathological process. Given the lack of effective preventive or treatment options against these diseases, the exploitation of new pharmacological agents is critically required.

**Method:** We assessed the efficacy of punicalin on receptor activator of nuclear factor-κB ligand (RANKL)-mediated osteoclast formation, F-actin ring formation, gene expression, bone resorption, nuclear factor-κB (NF-κB) as well as on mitogen-activated protein kinase (MAPK) signaling pathways and molecular docking *in vitro*. The impact of punicalin on breast cancer-induced osteoclastogenesis, breast cancer cell proliferation, and apoptosis were examined. Transwell assays were also performed. Moreover, we evaluated *in vivo* effects of punicalin in postmenopausal osteoporosis models and breast cancer bone metastasis model by micro-CT scanning and histomorphometry.

**Results:** Punicalin inhibited osteoclast formation, F-actin ring formation, bone resorption, as well as osteoclast-related gene expression by suppressing the NF-κB signaling pathway. *In vitro*, punicalin also suppressed the breast cancer-induced osteoclastogenesis, and proliferation, migration as well as invasion of MDA-MB-231 cells and dose-dependently promoted their apoptosis. *In vivo*, punicalin significantly suppressed breast cancer-induced osteolysis, breast cancer-associated bone metastasis, and ovariectomized (OVX)-mediated osteoporosis by repressing osteoclast and breast cancer cell.

**Conclusion:** Punicalin is expected to offer a novel treatment for the prevention of osteolysis diseases, including osteoporosis and breast cancer-associated osteolysis.

## Introduction

Globally, among women, the prevalence of breast cancer is the highest and breast cancer is one of the tumors with the highest morbidity and mortality rates (Jemal et al., 2007). The bone is prone to invasion by breast cancer, and nearly 75% of advanced breast cancer patient develop bone metastasis (Costa and Major 2009; Casas et al., 2013). Although bone metastases themselves rarely directly cause death in breast cancer individuals, serious complications of bone metastasis, such as hypercalcemia, chronic pain, incontinence and paralysis caused by spinal cord compression due to pathological fractures, all seriously affect life quality and breast cancer prognostic outcomes (Coleman, 1997; Coleman et al., 1998; Coleman, 2001). In recent years, progress in cancer treatment has increased life expectancy and, on the contrary, increased the risk of bone metastases (Nakai et al., 2019). There is still a lack of ideal therapeutic methods for breast cancer-associated bone metastasis. The current therapeutic approach involves surgical removal of the bone metastatic lesion in combination with drug therapy (Zhang et al., 2020). However, the incidence of perioperative complications is high, and the outcomes are not entirely satisfactory (Wegener et al., 2012).

During breast cancer bone metastasis, osteoclasts, rather than tumor cells, are responsible for osteolysis (Taube et al., 1994). Receptor activator of nuclear factor-κB ligand (RANKL), which belongs to the tumor necrosis factor (TNF) superfamily, is a vital role in differentiation of osteoclast in the bone microenvironment and is a competitor for osteoprotegerin (OPG) when binding receptor activator of nuclear factor-κB (RANK) on osteoclast precursor surfaces, leading to further induction of TRAF6 activation (Chen et al., 2018; Nakai et al., 2019). The downstream signaling pathway involved in TRAF6-induced osteoclast differentiation, comprising activation of nuclear factor-κB (NF-κB) as well as mitogen-activated protein kinase (MAPK) signaling pathways, upregulate expression levels of osteoclastogenesis-related transcription factors in the nucleus (He et al., 2014; Koga et al., 2019). Metastatic breast cancer cells cause increased expression of RANKL by releasing cytokines, which leads to overactivation of osteoclasts and pathological osteolysis (Roodman, 2004). Some cytokines are released during osteolysis, which enhance breast cancer cell growth, leading to a “vicious cycle” (Roodman, 2004). Therefore, activation of RANKL signaling pathway plays a central function in breast cancer bone metastasis. Blocking any link of this signaling pathway could be a possible therapeutic strategy for breast cancer-associated bone metastases, which can effectively prevent and cure osteolysis caused by breast cancer. Anti-osteoclast bisphosphonates and denosumab can slow down the progression of metastatic breast cancer-induced osteolysis (Stopeck et al., 2010; Van Poznak et al., 2011). Unfortunately, these drugs may cause adverse reactions, including renal impairment and jaw osteonecrosis. Bisphosphonates are not ideal for all breast cancer patients (Marx et al., 2005). In addition, anti-bone resorption treatment is palliative and does not inhibit the invasion of tumor cells. Chemotherapy can suppress cancer cell metastasis, however, the lack of anti-osteoclast activities is one of its defects (Valkenburg et al., 2013). Therefore, to inhibit breast cancer-induced osteolysis as well as bone metastasis, the current approach mainly uses combination therapy (Sun et al., 2019). Development of a drug with the ability to suppress both osteoclast differentiation as well as breast cancer progression will promote the treatment and prevention of breast cancer bone metastasis.

**FIGURE 1:**
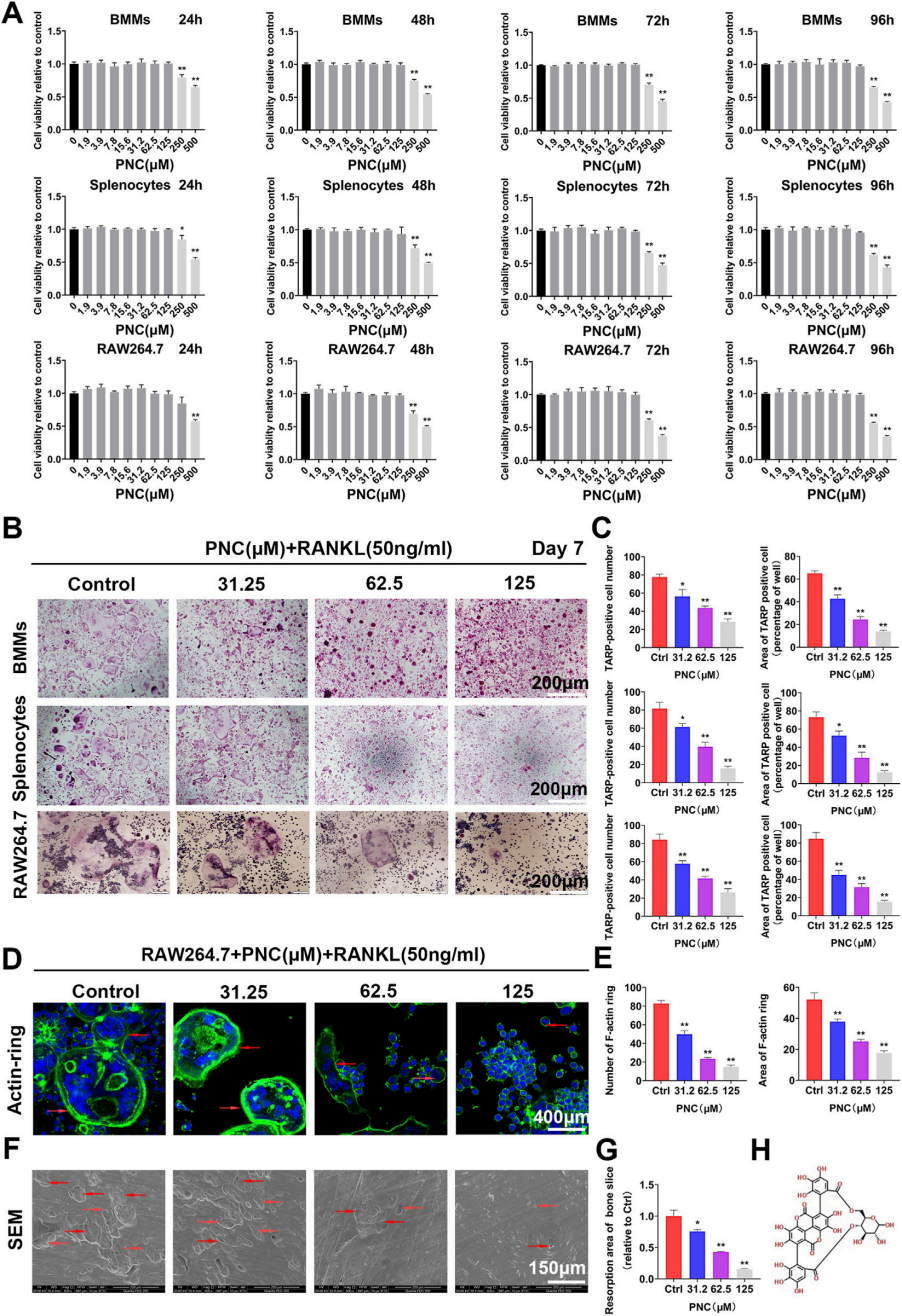
Nontoxic punicalin (PNC) inhibited RANKL-mediated osteoclast differentiation as well as function *in vitro*. **(A)** Cell viabilities of preosteoclasts after PNC treatments from 24 to 96 h (*n* = 3). **(B)** Osteoclastogenesis of three kinds of preosteoclasts, BMMs, splenocytes and RAW 264.7 cells in vitro after RANKL and PNC administration. **(C)** Quantification of osteoclast formation by PNC (*n* = 3). **(D)** F-actin ring formation after RANKL and PNC treatment. DAPI indicated cell nuclei (the red arrows indicate the F-actin ring). **(E)** Quantitative analysis of F-actin rings and osteoclast formation (*n* = 3). **(F)** Bone resorption pit formation after RANKL as well as PNC administration (the red arrows mark bone resorption pits). **(G)** Quantitative analyses of bone resorption pits (*n* = 3). **(H)** Structural formula of PNC. **p < 0.05, **p < 0.01 compared to controls

Punicalin (PNC, Figure 1H), one major ellagitannin (ET) in pomegranate peel, exerts many biological activities, including antioxidant (Wang et al., 2013), anti-inflammation (Lin et al., 1999), anti-hepatotoxic (Lin et al., 2001), anti-hepatitis B virus (Liu et al., 2016), anticancer (Heber 2008), anti-HIV replication (Martino et al., 2004), and antibacterial (Akiyama et al., 2001) properties. In addition, there is evidence that PNC inhibits activations of MAPK as well as NF-κB signaling pathways in human epidermal keratinocytes (Afaq et al., 2005), and the two pathways are tightly linked to osteoclast formation (Liu et al., 2019). Given the potential anticancer function and other potential therapeutic activities of PNC, we speculated that PNC may attenuate breast cancer bone metastasis and osteoclastogenesis.

We found that PNC suppresses RANKL-associated osteoclastogenesis (in BMMs, splenocytes, and RAW 264.7 cells), bone resorption, F-actin ring formation, as well as osteoclast-specific gene expressions by suppressing the NF-κB signaling pathways *in vitro*. PNC also inhibited MDA-MB-231 human breast cancer cell proliferation, migration, invasion and tumor cell-mediated osteoclast development. Moreover, it promoted their apoptosis *in vitro*. In ovariectomized (OVX) and breast cancer bone metastasis mouse models, treatment with PNC markedly prevented the breast cancer-induced osteolysis and OVX-mediated osteoporosis *in vivo*. Thus, PNC is a potential treatment option for osteoporosis and metastatic breast cancer-induced osteolysis.

## Materials and Methods

### Materials, Reagents and Cell Culture

PNC (>98% pure, Figure 1H) was acquired from Chengdu MUST Biotechnology (Sichuan, China) and dissolved in alpha minimum essential medium (a-MEM; Gaithersburg, MD, United States) to establish a 62.5 mM solution maintained at 4°C. Bovine bone slices we use are purchased from Shanghai Lushen Biotechnology Co., Ltd. Soluble mouse recombinant macrophage colony-stimulating factor (M-CSF), RANKL, fetal bovine serum (FBS), and penicillin as well as streptomycin were acquired from R&D Systems (Minneapolis, MN, United States). DMSO and TRAP staining kits were acquired from Sigma-Aldrich (St. Louis, MO, United States) while cell counting kits (CCK-8) were procured from KeyGen Biotech (Nanjing, China). The remaining reagents were bought from Sigma Aldrich, except if stated otherwise.

RAW 264.7 cells, an osteoclast precursor, were bought from American Type Culture Collection (ATCC; Rockville, MD, United States) and cultured in α-MEM medium with 1% penicillin/streptomycin and FBS (10%). For induction of osteoclast differentiation *in vitro*, genetically pure C57BL/6 female mice (4–6 weeks in age) were procured from Hunan Silaikejingda Experimental Animal Co., Ltd. (Changsha, China) and used to extract 1) primary splenocytes from ground spleen tissues and 2) primary bone marrow macrophages (BMMs) from tibias as well as femurs. The primary splenocytes as well as the BMMs were incubated in α-MEM with M-CSF (30 ng/ml). MDA-MB-231 cells were procured from the American Type Culture Collection (Rockville, MD, United States) and incubated in Dulbecco’s modified Eagle’s medium (DMEM) that had been supplemented with 10% FBS and antibiotics. All cells used in this study were kept under sterile conditions and a persistent high-humidity, 5% CO_2_ atmosphere at 37°C. Removal of non-adherent cells was done prior to each passage.

### Antibodies

Anti-rabbit IκBα (10268-1-AP), Anti-mouse JNK (66210-1-Ig), Anti-rabbit P38 (14064-1-AP), Anti-mouse β-actin (66009-1-Ig) and Anti-rabbit β-actin (20536-1-AP) were purchased from Proteintech (Chicago, United States); Anti-rabbit p-IκBα (ab133462), Anti-rabbit ERK (ab184699), Anti-rabbit p-JNK (ab179461) and Anti-rabbit p-P38 (ab47363) were purchased from Abcam (Cambridge, United Kingdom). Anti-rabbit p-ERK (4370s) and Anti-rabbit Cleaved Caspase-3 (Asp175) (5A1E) were purchased from CST (Boston, United States). Anti-rabbit Bcl-2 Rabbit (A0208) was purchased from ABclonal (Boston, United States).

### Cell Viability

We detected PNC-associated cytotoxic effects on three osteoclast precursor cell types (RAW 264.7 cells, splenocytes, BMMs) and MDA-MB-231 cell proliferation by conducting the CCK-8 assays. The three osteoclast precursor cell types (3 × 10^3^ cells/well) were incubated overnight in 96-well plates until adhesion. Twenty-four h after culture, cell viability was evaluated. Then, cells were subjected to diverse PNC concentrations (0, 1.9, 3.9, 7.8, 15.6, 31.2, 62.5, 125, 250, and 500 μM) for 24, 48, 72, or 96 h. Each well contained 100 μl of serum-free α-MEM (including CCK-8 reagent (10 μl)). Then, cells were incubated for 90 min. Measurement of absorbance was done using the ELX800 microplate reader (Abcam, United States) at 450 and 650 nm.

### *In Vitro* Osteoclastogenesis Assessment

To adequately investigate inhibitory effects of PNC on RANKL-mediated osteoclast formation, three osteoclast precursor cell types (RAW 264.7 cells, BMMs, and splenocytes, 3×10^3^ cells per well) were cultured in 96-well plates after which they were exposed to non-toxic PNC concentrations, ranging from 0 to 125 μM. Stimulation of preosteoclasts was done for up to 7 days using RANKL (50 ng/ml). For evaluation of PNC-mediated attenuation of MBA-MD-231 human breast cancer cell-mediated formation of osteoclasts, we first mixed RAW264.7 cells and MDA-MB-231 cells to contain 2 × 10^3^ RAW 264.7 cells and 1 × 10^3^ MDA-MB-231 cells per 100 ul. Then the complete medium containing two kinds of cells was dropped into the 96-well plate to 100 ul per well. Meanwhile, these cells are treated with PNC (0, 32.2, 62.5, or 125 μM), co-cultured for a total of 7 days after which TRAP staining was performed. After osteoclastogenesis, cell fixing was done in paraformaldehyde (PFA, 4%) and branded by staining with tartrate-resistant acid phosphatase (TRAP). In this study, osteoclasts with ≥3 nuclei were categorized as TRAP-positive, implying mature, differentiated multinucleated osteoclasts.

### Bone Absorption Pit Evaluation

Culture of RAW 264.7 cells (3,000 cells/slice) was done on sterile bovine bone slice surfaces in 96-well culture plates and subjected to PNC at diverse concentrations (from 0 to 125 µM). Furthermore, for osteoclast formation, RAW 264.7 cells were treated with RANKL (50 ng/ml). After the formation of mature osteoclasts, bone slice-adhered cells were eliminated through mechanical agitation as well as sonication. Finally, investigation of osteolytic absorption pits was done by scanning electron microscopy (SEM, FEI Quanta 250). Quantitative analyses (%) of resorbed bone surface areas were done by the ImageJ software (National Institutes of Health, Bethesda, MD, United States).

### F-Actin Ring Immunofluorescence Assay

F-actin formation was conducted. RANKL (50 ng/ml) as well as PNC at varying concentrations (0, 31.2, 62.5, and 125 μM) were used to treat RAW 264.7 cells, which were fixed for 15 min in paraformaldehyde (4%), and placed in 0.1% Triton X-100 (Sigma-Aldrich) for 5 min at room temperature after RAW264.7 cell differentiation. We cultured the cells in Alexa Fluor 647 phalloidin (Invitrogen, San Diego, CA, United States) in 0.2% (w/v) BSA-PBS (Invitrogen) to stain osteoclast cytoskeletons. Then, cells were washed using BSA-PBS (0.2% (w/v)) as well as PBS (phosphate-buffered saline), mounted using ProLong Gold anti-fade medium (Invitrogen). Observation and quantification of F-actin ring formation in each sample was done by confocal microscopy (Leica TCS SP8, Germany).

### Extraction of RNA and Quantitative Polymerase Chain Reaction Analysis

For analysis of osteoclast-specific gene expressions, inoculation of RAW 264.7 cells was done in a six-well plate and treated with PNC (0, 31.2, 62.5 or 125 μM). Then, total RNA extraction was conducted using the RNeasy Mini Kit (Qiagen, Valencia, CA, United States) as directed by the manufacturer. cDNA synthesis was done by a reverse transcriptase kit from TaKaRa Biotechnology, Japan. A SYBR Premix Ex Taq Kit (TaKaRa Biotechnology) and ABI 7500 Sequencing Detection System (Applied Biosystems; Foster City, CA, United States) were used for q-PCR analysis. Conditions for PCR were: 40 cycles of denaturation for 5 s at 95°C followed by amplification for 34 s at 60°C. Experiments were conducted in triplicates. Primer sequences are shown in Table 1.

**TABLE 1:** Quantitative polymerase chain reaction (q-PCR) analysis of mouse primer sequences.

Gene	Forward primer (5′-3′)	Reverse primer (5′-3′)
c-fos	CCA​GTC​AAG​AGC​ATC​AGC​AA	AAG​TAG​TGC​AGC​CCG​GAG​TA
V-ATPase-d2	AAG​CCT​TTG​TTT​GAC​GCT​GT	TTC​GAT​GCC​TCT​GTG​AGA​TG
Cathepsin-K	CTT​CCA​ATA​CGT​GCA​GCA​GA	TCT​TCA​GGG​CTT​TCT​CGT​TC
CTR	TGC​AGA​CAA​CTC​TTG​GTT​GG	TCG​GTT​TCT​TCT​CCT​CTG​GA
TRAP	CTG​GAG​TGC​ACG​ATG​CCA​GCG​ACA	TCC​GTG​CTC​GGC​GAT​GGA​CCA​GA
GAPDH	ACC​CAG​AAG​ACT​GTG​GAT​GG	CAC​ATT​GGG​GGT​AGG​AAC​AC
NFATc1	CCG​TTG​CTT​CCA​GAA​AAT​AAC​A	TGT​GGG​ATG​TGA​ACT​CGG​AA

### Western Blotting

To evaluate the impact of PNC on signaling pathways, RAW 264.7 cells that had been inoculated in 6-well plates (5 × 10^5^ cells/well) in complete α-MEM supplemented with 1% streptomycin/penicillin, 10% FBS were incubated. After verification of the appearance of osteoclasts, cells were incubated for 4 h with or without 125 μM PNC treatment followed by incubation with RANKL (50 ng/ml) for 0, 5, 10, 20, 30, or 60 min. The medium was removed and rinsed twice with phosphate buffer. Radioimmunoprecipitation assay (RIPA) lysis buffer (Well Biology, Changsha, China) and a protease inhibitor cocktail were used to extract total proteins from the cultured cells. Then, centrifugation of the lysate was done for 15 min at 12,000 rpm after which the supernatant with the protein was obtained. Measurement of protein concentrations was done using the bicinchoninic acid (BCA, Thermo Fisher, MA, United States) assay.

A specific amount of every cell lysate (30 mg) was dissolved in sodium dodecyl sulfate polyacrylamide gel (10%). Products were transferred to polyvinylidene difluoride membranes (Millipore; Bedford, MA, United States) that were later blocked for 2 h using 5% non-fat milk powder in TBS-Tween (TBS: 0.05 M Tris, 0.2% Tween-20, 0.15 M NaCl, and pH 7.5). Overnight incubation was done at 4°C in the presence of primary antibodies. Then, TBS-Tween was used to wash the membranes followed by incubation for 2 h in the presence of IRDye 800CW (molecular weight = 1,162 Da)-conjugated secondary antibodies.

Culturing of MDA-MB-231 cells was done in 6-well plates (density: 5 × 10^5^ cells/well) with DMEM, antibiotics as well as 10% FBS. For 2 days, they were treated with PNC (0, 62.5, or 125 μM). Following treatment, C-caspase-3 activities were measured by the Caspase Colorimetric Assay Kit (KeyGen Biotech) as instructed by the manufacturer. Briefly, cell lysates (about 0.1 mg total protein) were supplemented to the reaction mixture, including caspase-3 colorimetric substrate peptides. Incubation for 4 h was done at 37°C. Finally, visualization of proteins on the blots was done by an Odyssey infrared imaging system (LI-COR, Nebraska, United States). Band intensities were measured and quantified using ImageJ software.

### NF-κB Luciferase Reporter Gene Assay

For determination of if PNC disturbed NF-κB signaling pathway activation in RAW 264.7 cells, an NF-κB luciferase reporter construct was transfected in cells. RAW 264.7 cells (1 × 10^5^ cells per well) were inoculated in a 24-well plate, followed by 24 h of incubation and treated for 1 h with PNC (0, 31.2, 62.5, or 125 μM) prior to incubation with RANKL (50 ng/ml). Then, cell lysates were incubated for 2 min at room temperature using substrate (Promega, Madison, WI, United States). We used the Promega Luciferase Assay System (Promega) to measure luciferase activities as instructed by the manufacturer. The control group was used to normalize luciferase activities. Experiments were done in triplicates.

### Molecular Docking Analyais

Using the structure of IκBα as a template, mouse IκBα kinase domain homology models were constructed using Modeler 9.12. Based on AutoDock and AutoDock Vina (Trott and Olson, 2010; Ouyang et al., 2019), PROCHECK was utilized to demonstrate stereochemical architectures of IκBα, while creation of the link between IκBα and kinases was done using the Lamarckian genetic algorithm. Molecular docking figures showing binding were established using a PyMOL Visualization Software (Schrödinger LLC, New York, NY, United States).

### Micro-CT Scanning

After euthanasia of the mice treated with PNC, the tibia and fibula were obtained and fixed for 48 h in 4% paraformaldehyde. Then, tibia and fibula of the mice were analyzed by high-resolution micro-CT. The scanning parameters were: minimum resolution of 10 μM, 80 kV scanning voltage, and scanning current of 80 mA (Ouyang et al., 2019). After the region of interest in the proximal tibia near the tibial plateau was measured, the data were obtained, including relevant bone surface/volume ratio (BS/BV), bone mineral density (BMD), trabecular number (Tb.N), and trabecular space (Tb.Sp), bone volume/tissue volume (BV/TV), trabecular thickness (Tb.Th).

### Histological and Histomorphometric Analyses

Mouse tibia and femur samples undergoing micro-CT scanning analysis were decalcified for 5 weeks using 10% EDTA and embedded in paraffin. Tissue sections were used for TRAP as well as H&E staining. High-resolution microscopy was performed for imaging and specimen examination. Finally, the abundance of TRAP-positive multinucleated osteoclasts in each specimen was determined. The ratio of the trabecular osteoclast number to bone surface (Oc.N/BS, N/mm) and the percentage of osteoclast surface to bone surface (Oc.S/BS,%) were measured on trap stained sections at 200-fold magnification (Sakata et al., 1999). Under microscopy (Leica image analysis system, Q500MC), ImageJ (NIH, United States) was used for bone static histomorphometric evaluation of tumor area, Oc.N/BS and Oc.S/BS.

### Apoptosis Assay

PNC-mediated apoptotic outcomes on MDA-MB-231 cells were assayed by the Vybrant Apoptosis Assay Kit #2 (Invitrogen). After treatment with PNC (0, 62.5, 125, and 250 μM) for 48 h, MDA-MB-231 cells were rinsed twice using sterile PBS after which they were pelleted. Supernatants remained unused and were discarded while cells resuspended in 1X Annexin-binding buffer. To evaluate early apoptosis, cell staining was done using propidium iodide and Alexa Fluor 488 Annexin V using the Vybrant Apoptosis Assay Kit #2 (Invitrogen). Fluorescence-activated cell sorting (FACS) was carried out by FACScan flow cytometry (Becton-Dickinson, Sunnyvale, CA, United States). Data were obtained using the CELL Quest software.

### Invasion and Migration Assay

BioCoat™ Matrigel™ Invasion Chambers (BD Biosciences) with 24-well chambers and pore polycarbonate filters (8-µm) as well as Transwell^®^ permeable supports (Corning, Inc., Acton, MA, United States) with 24-well chambers and pore polycarbonate filters (8-µm) were utilized in invasion and migration assays as instructed by the manufacturers. The MDA-MB-231 cells (4 × 10^4^) were seeded in serum-free medium (100 µl) with varying concentrations of PNC (0, 62.5, 125, and 250 µM), and 500 µl of complete medium was seeded in the lower wells. After cultivation for 24 h, MDA-MB-231 cells in the top chambers were eliminated using a cotton swab. Invading and migrating cells at the bottom of the filter were fixed for 30 min in paraformaldehyde (4%) and stained for 6 min using crystal violet (1%). Pictures were obtained by Olympus inverted microscopy. Invaded and migrated MDA-MB-231 cells were counted by ImageJ software.

### Ovariectomized Mouse Model

Animal experimental procedures involved in this study were all permitted by the Animal Care Committee of Central South University. Female C57BL/6 mice (8 weeks old, female, *n* = 30) used in the experiment were acquired from Hunan Silaikejingda Experimental Animal Co., Ltd., (Changsha, China). The mice were placed in plastic cages in specific pathogen-free (SPF) conditions for 1 week to acclimate to the new environment before undergoing OVX surgery. Mice (*n* = 20) were anaesthetized by intraperitoneal injection of pentobarbital (40 mg/kg, Sinopharm Shanghai Co., Ltd.) preoperatively, followed by bilateral ovariectomy (Ouyang et al., 2019). After OVX resection, mice were randomly divided into three groups: the sham non-OVX mice, the vehicle-treated OVX mice, PNC-treated OVX mice administered with 5 mg/kg/day (PNC intraperitoneally), with *n* = 10 per group. Concentrations of PNC were determined after a preliminary screening *in vivo* by referring to other studies (Lin et al., 1999). Sham and vehicle mice were injected with sterile PBS. The injected mice that were closely observed for 4 weeks were euthanized at the end of the experiments. Finally, the right tibia of the mice was harvested for micro-CT scanning.

### Intratibial Xenograft Model of Breast Cancer Bone Metastasis

Female BALB/c-nu/nu mice (6 weeks old, *n* = 30) used in the protocol were acquired from Hunan Silaikejingda Experimental Animal Co., Ltd., (Changsha, China) and kept in plastic cages under pathogen-free (SPF) environments for a week. After acclimating to the new facility and environment, the mice involved in the study were randomized into three groups: placebo control group (sterile PBS, *n* = 10), vehicle group (sterile PBS, *n* = 10), and PNC group (5 mg/kg/day, intraperitoneally, *n* = 10). The concentration of PNC used was determined after a preliminary screening *in vivo*. Sham and vehicle mice were injected with sterile PBS for 28 days. The PNC-treated and vehicle-treated mice were administered with MDA-MB-231 (10^7^ cells/ml) in the tibial plateau of the right leg to construct a bone metastasis model of breast cancer. Finally, injected mice were nurtured for 28 days and euthanized, and the right tibia of the mice was harvested for micro-CT scanning.

### Statistical Calculations

All experiments were separately carried out in replicates of three or more. Data are shown as mean ± SD. Determination of significance was done by the Student’s *t*-test in SPSS 22.0 software (SPSS, Inc., United States). *p* < 0.05 was considered significant.

## Results

### Punicalin Suppressed Osteoclast Function and Formation at Noncytotoxic Concentrations *In Vitro*

To confirm PNC-associated effects on osteoclasts rather than a toxic effect, we first determined the nontoxic concentrations of PNC against preosteoclasts through the cell counting kit-8 (CCK-8) test. Figure 1A shows that there were no cytotoxic effects on the three osteoclast precursor cell types (RAW 264.7 cells, BMMs, and splenocytes) when PNC concentrations were ≤125 μM. Based on the above experimental results, nontoxic concentrations of 31.2, 62.5, and 125 μM were selected to carry out the following anti-osteoclastogenesis experiments. We observed that, in the control group, despite significant osteoclastogenesis showing round, large, and red-stained multinucleated osteoclasts, addition of PNC dose-dependently diminished the area and number of the mature TRAP-positive osteoclasts from RAW 264.7 cells, BMMs, and splenocytes (Figures 1B,C), indicating that a nontoxic concentration of PNC could inhibit osteoclastogenesis.

In addition, well-shaped F-actin rings are essential for osteoclast function, so we investigated the impact of PNC treatment on the formation of F-actins. In the control group, RAW 264.7 cells produced polarized actin rings after RANKL stimulation, while PNC treatment decreased the number and size of the F-actin rings, suggesting that PNC influenced osteoclast function (Figures 1D,E). This finding is consistent with osteolysis on bone slices as detected by scanning electron microscopy (Figures 1F,G), showing that deep and big bone resorption pits were present in RANKL stimulated control group. The addition of PNC reduced the size and number of bone resorption pits. The above results indicate that the function and formation of osteoclasts are effectively inhibited by nontoxic concentrations of PNC and encouraged us to investigate potential effects of PNC on the formation of osteoclasts.

### Punicalin Inhibited Osteoclast-Specific Gene Expression Levels *In Vitro*

**FIGURE 2:**
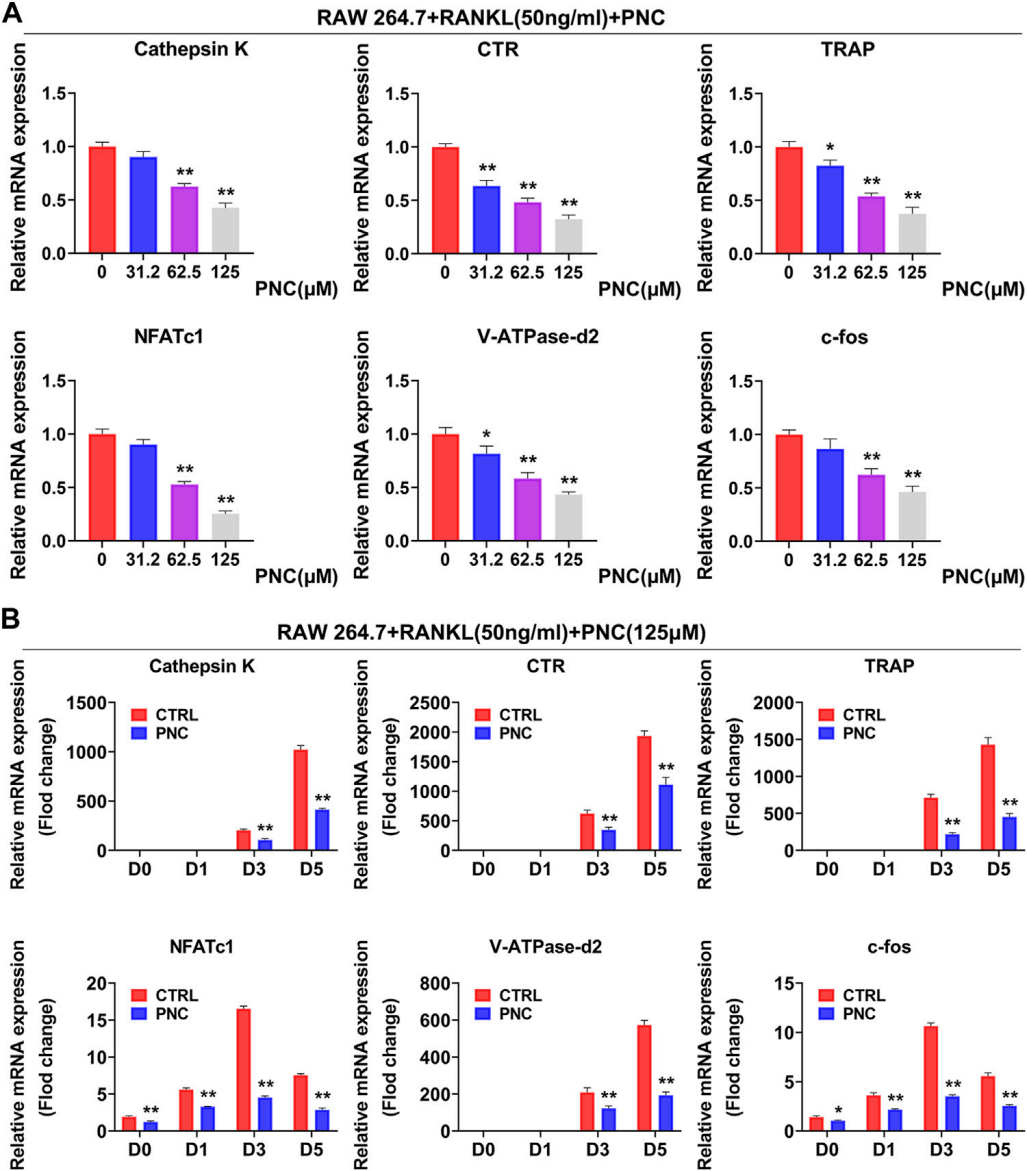
PNC impaired RANKL-mediated expressions of specific genes in osteoclastogenesis. **(A)** Diverse concentration of PNC (0, 32.2, 62.5, 125 μM) mitigated the transcription of osteoclast-specific genes, including CTR, Cathepsin K, TRAP, V-ATPase-d2, NFATc1, and c-fos. **(B)** with or without 125 μM PNC, for 0, 1, 3, or 5 days separately. Expressions of specific genes associated in osteoclast formation were determined through real-time PCR and normalized to β-actin. **p < 0.05, **p < 0.01 compared to the control group.

Expressions of the specific genes involved in differentiation of osteoclast were activated after stimulation using RANKL. The effect of PNC on the formation of osteoclasts was done by examining RANKL-induced mRNA expressions of Calcitonin receptor (CTR), NFATc1, TRAP, Cathepsin K, V-ATPase-d2, and c-fos. In the control group, expressions of all genes were induced by RANKL, while PNC treatment time- and dose-dependently downregulated osteoclast gene expression (Figures 2A,B). The above experimental data further demonstrated that PNC at nontoxic concentrations inhibited specific gene expressions in osteoclast formation *in vitro*.

### PNC Inhibited RANKL-Associated NF-κB Activation During Osteoclast Differentiation

To investigate specific inhibitory effects of PNC on osteoclasts, we evaluated two important pathways involved in the formation of osteoclasts, namely NF-κB and MAPK signaling pathways (Hu et al., 2020). Therefore, we tried to establish the impact of PNC on expressions of NF-κB as well as MAPK signaling pathways through Western blots to confirm the mechanism through which PNC suppresses osteoclastogenesis.

**FIGURE 3:**
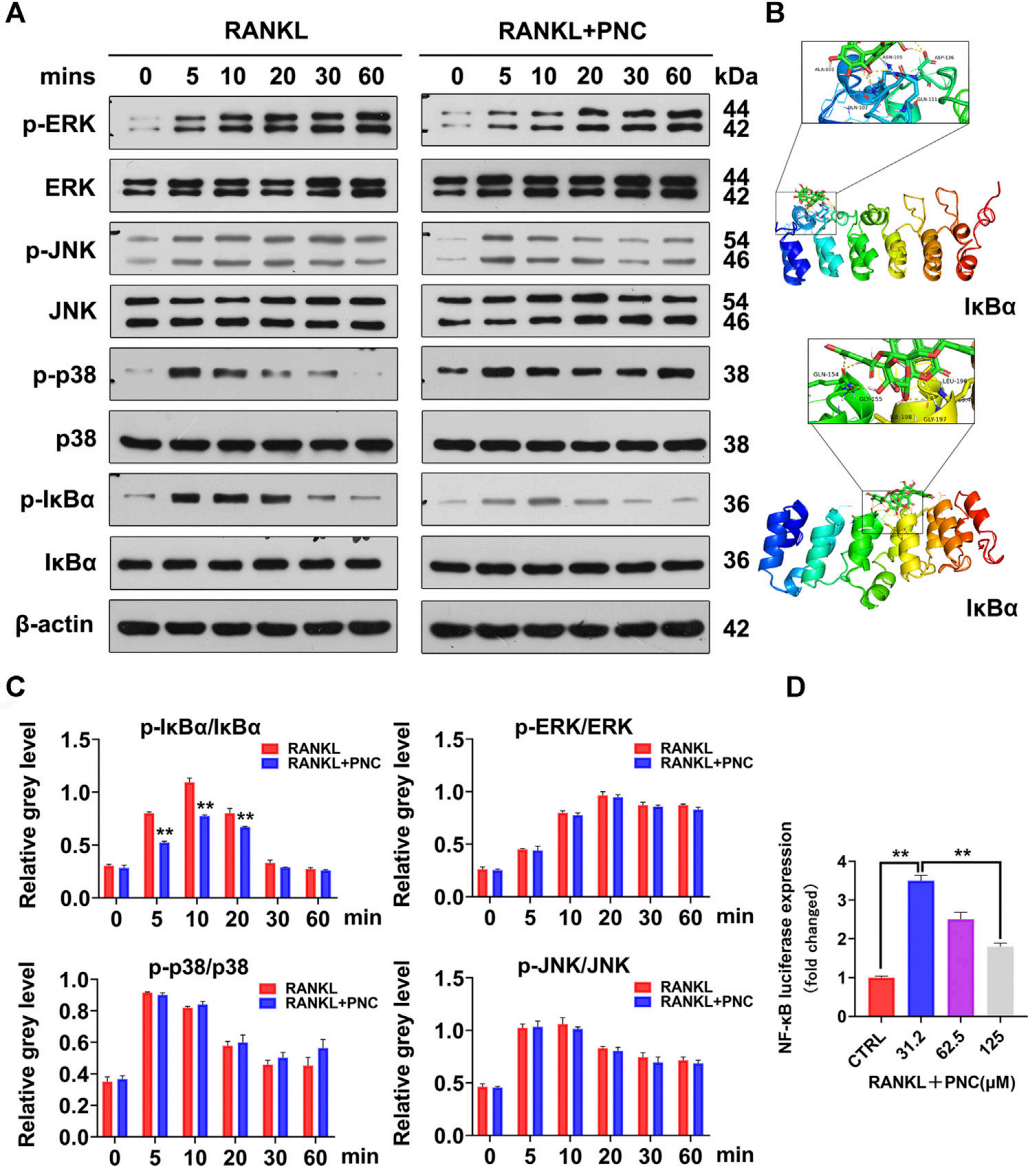
PNC suppressed RANKL-initiated activation of the NF-κB signaling pathway. **(A)** Assessment of the inhibitory effects of PNC on RANKL-mediated NF-κB and MAPK signaling. **(B)** Molecular docking of PNC with IκBα kinases. **(C)** Phosphorylated IκBa relative to IκBa, phosphorylated JNK relative to JNK, phosphorylated ERK relative to ERK, phosphorylated p38 relative to p38. **(D)** After culture with PNC and RANKL, the NF-κB luciferase activity of the transfected RAW 264.7 cells were assessed. **p < 0.05, **p < 0.01 versus the control group.

As shown in Figures 3A,C, the addition of PNC increased IκBα expression and reduced the ratio of phosphorylated IκBα compared with those in the RANKL stimulation group. IκBα is used to stabilize NF-κB. This molecule binds to cytoplasmic p65 of NF-κB, allowing NF-κB to remain stable (Hu et al., 2020). Therefore, the NF-κB pathway was inhibited by PNC treatment. From the above experimental results, we determined the probable binding sites between PNC and IκBα by molecular docking. As expected, PNC established molecular bonds with multiple amino acids, including Ala-102, Gln-107, Gln-111, Asp-136, Asn-105, Gln-154, Gly-155, Leu196, Gly197, and Ile-198 (Figure 3B). These findings imply that PNC may suppress RANKL-mediated NF-κB activation, thereby reducing the formation of osteoclasts. The inhibitory impacts of PNC on the NF-κB signaling pathways were confirmed by NF-κB luciferase reporter gene analysis (Figure 3D). As shown in Figure 3A, extracellular signal-regulated kinase (ERK) as well as c-Jun N-terminal kinase (JNK) signaling pathways seemed to be partially suppressed, and the p38 signaling pathways seemed to be partially activated, but the results obtained through statistical analysis were not statistically significant (Figure 3C). In summary, PNC suppresses the downstream NF-κB signaling pathway of RANKL by targeting IκBα binding during osteoclast formation.

### Punicalin Attenuated Bone Loss by Suppressing Osteoclast Formation in Ovariectomized Mice

**FIGURE 4:**
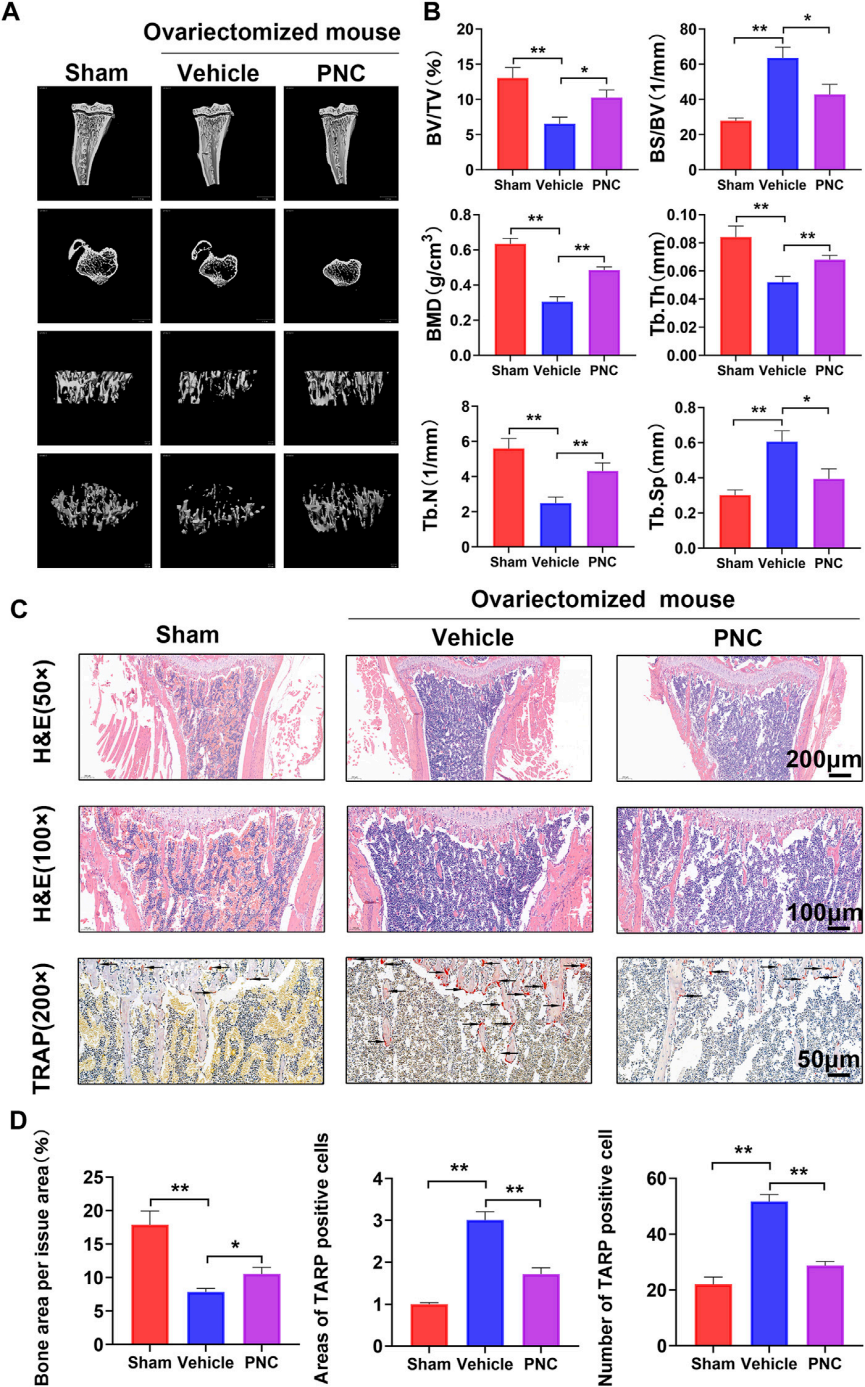
PNC attenuated bone resorption and suppressed osteoclast activation from the OVX mice. **(A)** High-resolution micro-CT scanning of tibia in OVX mice. Severe tibial trabecular destruction and bone resorption of the vehicle-treated mice compared to the PNC-treated OVX mice. **(B)** BS/BV, BV/TV, Tb.Th, Tb.N, BMD, and Tb.Sp of each tibia sample were determined as indicated in the methods (*n* = 5). **(C)** H&E and TRAP staining of tibial slices of the OVX mice (the black arrows of TRAP staining indicate TRAP-positive osteoclasts). **(D)** Area as well as number of TRAP-positive osteoclasts and bone area per tissue area were determined by ImageJ software. (*n* = 5). PNC = (5 mg/kg/day). **p < 0.05, **p < 0.01 versus the vehicle group.

We created an OVX mouse model to investigate suppressive effects of PNC on osteoclasts *in vivo*. Through Micro-CT scanning, it was revealed compared to the sham group, osteoporosis was found in proximal tibia of the vehicle group, with severe bone loss as well as incomplete bone trabecular structure (Figure 4A). In contrast, a slight bone loss as well as complete trabecular structures were observed in the PNC treatment group. Subsequently, we analyzed the bone parameters of bone trabeculae specimens in the proximal tibia, and we found that compared to the vehicle group, trabecular number (Tb.N.), bone mineral density (BMD), trabecular thickness (Tb.Th.), as well as bone volume/tissue volume ratio (BV/TV) were markedly elevated in the PNC treatment group, while trabecular spacing (Tb.Sp) and bone surface/volume ratio (BS/BV) was decreased (Figure 4B). The osteoprotective effect of PNC was further confirmed by histological morphological analysis. Figures 4C,D shows that TRAP positive cells in the vehicle group were increased in number and area, with severe trabecular structure impairment and obvious bone loss. In the PNC treatment group, the number as well as osteoclast areas were significantly reduced, and bone resorption were less severe, which was comparable to micro-CT scan results. According to the above results, PNC effectively alleviated bone loss by inhibiting osteoclasts in OVX mice.

### Punicalin Inhibited Breast Cancer Cell Proliferation, Migration, Invasion, Breast Cancer-Mediated Osteoclast Differentiation, and Promoted the Apoptosis of Breast Cancer Cells

**FIGURE 5:**
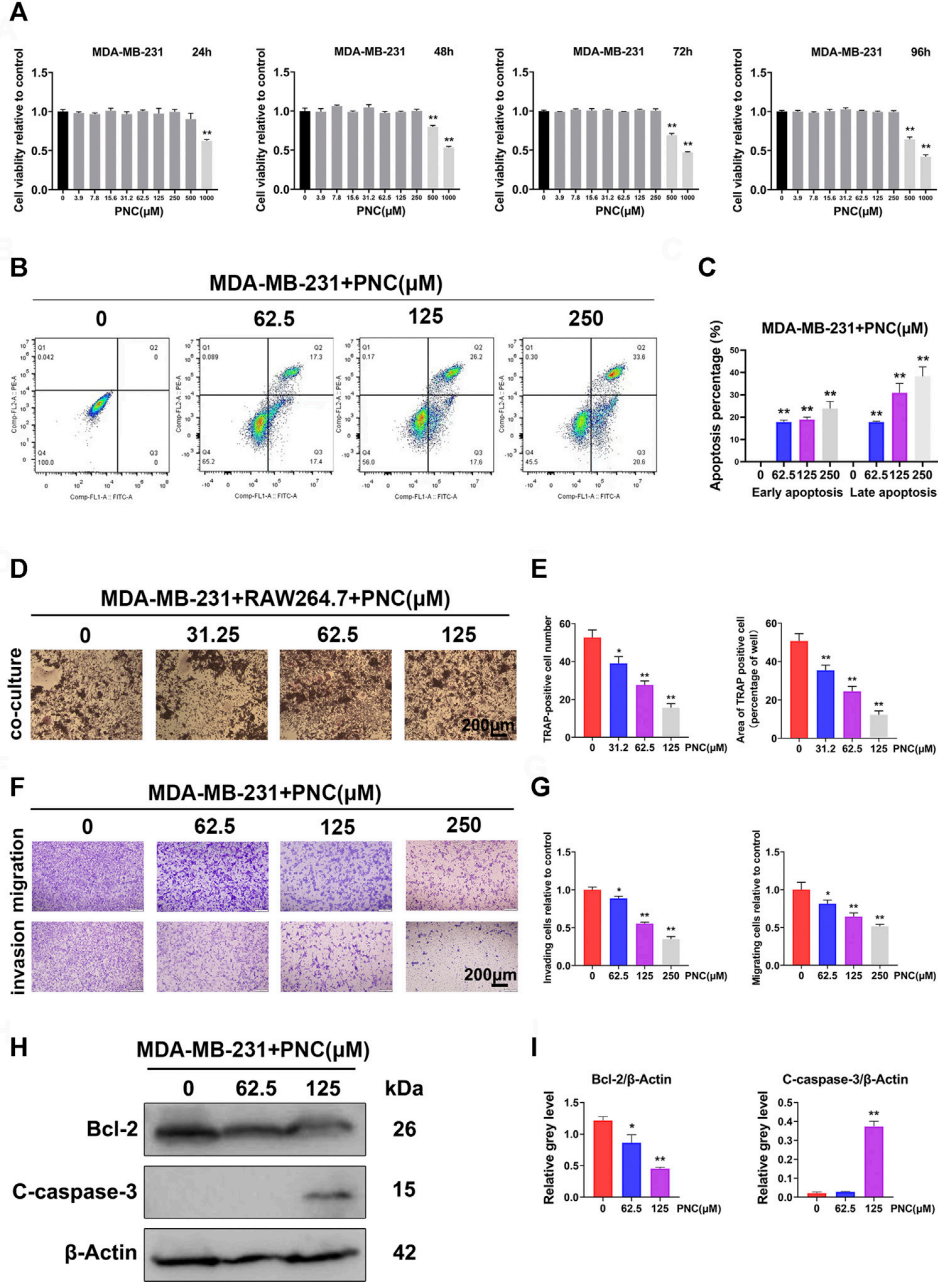
Nontoxic PNC suppressed human breast cancer MBA-MD-231 cell-induced osteoclastogenesis, promoted apoptosis of MBA-MD-231 cells, and inhibited the migration as well as invasion of MBA-MD-231 cells *in vitro*. **(A)** Viability of MBA-MD-231 cells after PNC treatments for 24–96 h (*n* = 3). **(B)** Flow cytometric analysis of apoptotic breast cancer cells with PNC treatment. **(C)** Quantification of the percentage of apoptotic MDA-MB-231 cells after treatment with diverse concentrations of PNC (0, 62.5, 125,250 μM) for 48 h (*n* = 3). **(D)** RAW 264.7 cells cocultured with MDA-MB-231 cells, treated with PNC, and cocultured for 1 week before TRAP staining. **(E)** Quantitative analysis of TRAP positive osteoclasts (*n* = 3). **(F)** Efficacy of PNC on the suppression of migration as well as invasion of MDA-MB-231 cells. The irregularly shaped cells, that invaded and migrated, showing purple staining were counted and photographed. **(G)** Quantification of the percentage of cell invasion and migration by ImageJ software. Bars represent the SD, whereas columns represent the means (*n* = 3). **(H)** After the PNC-treated MDA-MB-231 cells were cultured for 2 days, PNC-induced cleaved caspase-3 and Bcl-2 levels were examined using Western blotting. **(I)** Average ratios of cleaved caspase-3 as well as Bcl-2 relative to β-actin. **p < 0.05, **p < 0.01, when compared to the control group.

Figure 5A shows that after PNC treatment for 24 h, only PNC at concentrations of 1,000 μM showed toxic effects on tumor cells, but with increasing culture time, both 500 and 1,000 μM PNC showed toxic effects. This finding provides a potential range of options for us to delineate the therapeutic effect of PNC in breast cancer. Figures 5B,C shows that the early apoptotic effect of 62.5 μM PNC on MDA-MB-231 cells was observed compared to the control group. Moreover, MDA-MB-231 cell early apoptosis were elevated at higher concentrations of PNC (125 and 250 μM). The effect on late apoptosis was also observed after PNC treatment. The above studies confirmed that PNC at a nontoxic concentration can effectively promote human breast cancer cell apoptosis. To further study whether PNC has suppressive effects on osteoclast differentiation induced by MDA-MB-231 cells, we cocultured MDA-MB-231 cells and RAW 264.7 cells. Figures 5D,E shows that in the control group without PNC, after co-culture in the presence of MDA-MB-231 cells, the RAW 264.7 cells differentiated into osteoclasts. In contrast, after 31.2 μM PNC treatment, the induction effect was significantly inhibited, and the abundance of TRAP-positive cells was suppressed. With increasing PNC concentrations, inhibitory effects became more obvious. According to the microscopic osteoclast count, significant differences occurred between the control and treatment groups at the three concentrations of PNC, 31.2, 62.5 and 125 μM. From the above experiments, inhibitory effects of PNC on osteoclast differentiation induced by MDA-MB-231 cells are attributed to the combined effect of PNC on the inhibition of osteoclast formation and PNC on the promotion of apoptosis of MDA-MB-231 cells.

An important distinguishing features of malignant tumor cells compared to benign tumor cells is that the former shows strong migration as well as invasion. Therefore, we studied the impact of PNC on tumor cells. Figures 5F,G shows that MDA-MB-231 cells in the control group without PNC treatment showed strong invasion and migration. However, in the experimental group supplemented with PNC, 62.5 μM PNC had a certain inhibitory effect MDA-MB-231 cell invasion. As PNC concentrations increased, MDA-MB-231 cell invasions were significantly inhibited. Suppressive effect of PNC on MDA-MB-231 cell migration was similar to that of invasion.

To illustrate the underlying mechanisms of PNC-mediated apoptosis, we investigated the functions of antiapoptotic Bcl-2 protein, an important Bcl-2 family member (Cory and Adams, 2002). Protein expression levels of Bcl-2 were decreased after PNC treatment at concentrations of 62.5 and 125 μM (Figures 5H,I). Thus, we concluded that PNC modulates Bcl-2 expressions in MDA-MB-231 cells. Caspase-3 is a terminal executor of apoptosis belonging to the caspase cascade. Once cleaved, caspase-3 activates and triggers programming cell death (Emoto et al., 1995; Xue et al., 1996; Porter and Jänicke, 1999). To establish the function of the caspase-3 cascade in PNC-mediated apoptosis, we evaluated cleaved caspase-3 (active form of caspase-3) levels by western blots after PNC treatment. Figures 5H,I shows that PNC increased caspase-3 cleavage at 125 μM, while the lower concentration groups (62.5 μM) had no significant increase compared to the control group. Thus, in our study, the abundance of apoptotic cells was found to be increased after cells had been treated with 62.5 and 125 μM PNC.

### Punicalin Prevented Osteolysis in MDA-MB-231 Breast Cancer Cell-Derived Tumor-Bearing Mice *In Vivo*

**FIGURE 6:**
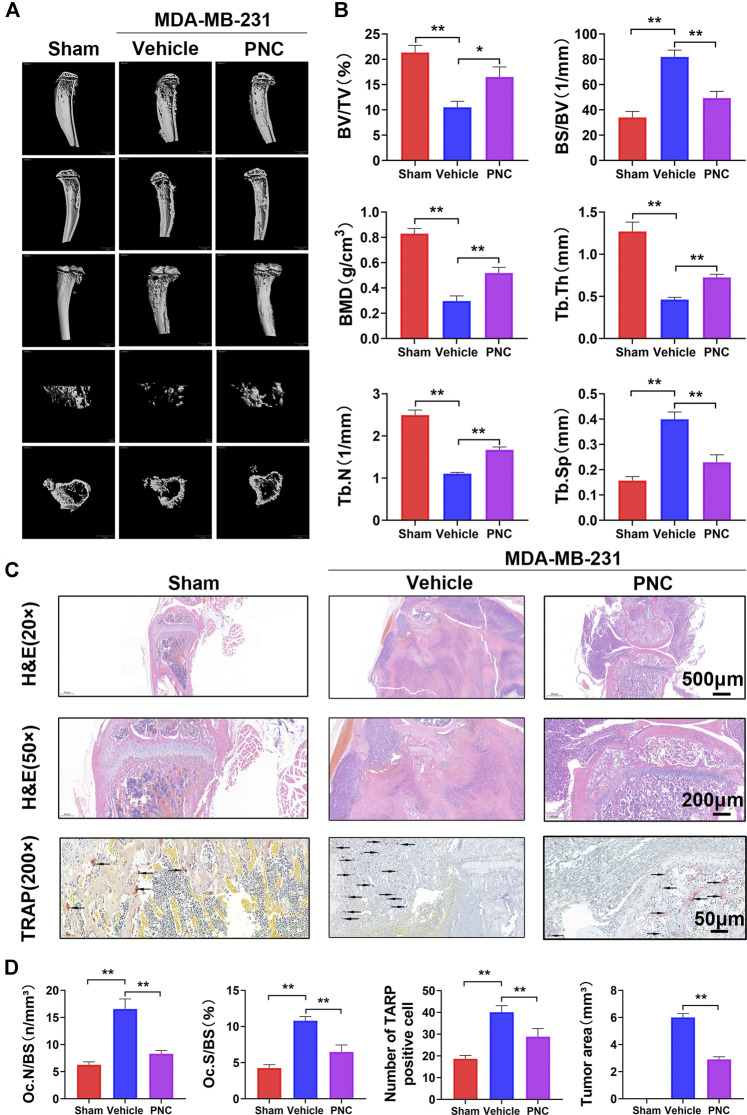
PNC reduced osteolysis and bone destruction in MDA-MB-231 cell-bearing mice. **(A)** Illustrative 3D computer reconstruction of the bone near the knee joint by micro-CT scanning exhibited extensive trabecular/cancellous bone loss of the tumor-bearing mice in the vehicle group. Treatment with PNC reduced bone loss in tumor-bearing mice. **(B)** Quantification of the bone parameters demonstrated that MDA-MB-231 tumor cells induced bone loss with significantly decreased Tb.N, BV/TV, BMD, and Tb.Th values as well as increased BS/BV and Tb.Sp. PNC rescued these bone parameters (*n* = 5). **(C)** TRAP and H&E staining of tibia slices from tumor-bearing mice. In the vehicle group, tumor-bearing mice showed severe osteolysis with discrete cortical bones. Some breast cancer cells even damage part of the metaphysis and cause tumor growth in the knee cavity. Osteoclasts positive for TRAP were observed at junction areas between tumor tissues and the bone. The addition of PNC retained the complete metaphysis and intact bone cortex, and the tumor grew locally in the bone marrow cavity; moreover, the number of TRAP-positive cells was lower in the PNC-treated mice (the black arrows of TRAP staining indicate TRAP-positive osteoclasts). **(D)** Quantification of the TRAP-positive osteoclasts, Oc.N/BS and Oc.S/BS determined using ImageJ software. All experiments were performed at least thrice (*n* = 5). PNC = (5 mg/kg/day). **p < 0.05, **p < 0.01 when compared to the vehicle group.

After the dual activity of osteoclast differentiation and tumor metastasis was inhibited by PNC *in vitro*, we further studied murine models of breast cancer bone metastases to establish the therapeutic effects of PNC. MDA-MB-231 cells were inoculated from the tibial plateau into vehicle-treated or PNC-treated nude mice to create a model of breast cancer bone metastasis. Four weeks after the injection of PNC (5 mg/kg/day), we performed micro-CT scanning near the proximal tibia of the mice and found severe bone erosion in the proximal tibia of tumor-bearing mice of the vehicle group without PNC treatment, while PNC treatment significantly relieved the osteolytic lesions near the proximal tibia (Figure 6A). Next, bone parameters, such as BV/TV, Tb.Sp, BMD, Tb.Th., BS/BV as well as Tb.N., were analyzed (Figure 6B). Compared to the vehicle group, bone mass in the PNC treatment group was significantly higher, while the BS/BV and Tb.Sp values of the vehicle group were higher. The dual activity of PNC in inhibiting osteoclast differentiation and tumor metastasis was further demonstrated by histological section staining. Figure 6C shows that bone cortex of proximal tibia of the tumor-bearing mice in the vehicle group was traversed by tumors, and the metaphysis was also damaged, which led to tumor invasion into the luminal growth of the knee joint. The addition of PNC protected the metaphyseal and cortical integrity and prevented the tumor from growing into the articular cavity of the knee joint. TRAP staining showed that compared with vehicle-treatment, PNC treatment significantly reduced the number of osteoclasts. Bone morphologic analysis of the proximal tibia, including the tumor area, Oc.N/BS and Oc.S/BS, further confirmed that PNC prevented breast cancer-induced pathological bone destruction (Figure 6D).

## Discussion

We determined that PNC can suppress RANKL and tumor-mediated osteoclastogenesis, suppressed breast cancer cell migration, invasion, and proliferation, induce their apoptosis, and exert a systemic and local anti-osteoclast effect *in vivo*, which is mainly mediated through the NF-κB signaling pathway. Therefore, the application of PNC might be a novel approach for preventing and treating osteolysis-associated diseases, such as osteoporosis and metastatic breast cancer-induced osteolysis.

To rule out drug toxicity effects on osteoclasts, we first tried to find a safe concentration of PNC. Studies have shown that PNC is safe at concentrations of 0–100 μM and has a slight inhibitory effect on mouse mononuclear macrophage J774A.1 cells at 150 μM (Shen et al., 2021). Consistent with the results, our study found that the nontoxicity concentration of PNC on preosteoclasts (BMMs, RAW 264.7 cells, and splenocytes) was lower than 125 μM (Figure 1A). However, the effects of PNC on differentiation of osteoclasts has not been clearly established. Therefore, we focused on its effect on the formation of osteoclasts. *In vitro*, PNC inhibited RANKL-mediated osteoclast formation as well as bone resorption.

To confirm the suppressive impact of PNC on the differentiation of osteoclasts, we studied the specific genes and signaling pathways related to osteoclasts. As major regulators of osteoclast differentiation, NFATc1 and c-fos led to expressions of osteoclast-associated genes, including V-ATPase-d2, Cathepsin K, TRAP, and CTR, which are also involved in bone resorption activities of osteoclasts (Boyle et al., 2003; Crotti et al., 2006; Yavropoulou and Yovos, 2008). Expression levels of these genes were suppressed. In addition, the process of osteoclast formation involves NF-κB as well as MAPK signaling pathways activation, the latter of which includes JNK, ERK, and p38 (Hu et al., 2020). After stimulation with RANKL, extracellular stimulation was transduced to the nucleus through NF-κB, ERK, JNK, and p38 phosphorylation, which led to differentiation into mature osteoclasts. It has been reported that PNC has suppressive effects on both NF-κB as well as MAPK signaling pathways (Afaq et al., 2005). We found that PNC suppresses NF-κB signaling pathway but has a minimal impact on MAPK signaling pathways. This difference may be due to different cell lines. A luciferase assay confirmed the suppressive effects of PNC on NF-κB signaling pathway. Then, we identified the underlying binding sites between PNC and NF-κB pathway through molecular docking analysis. It was found that PNC was embedded in the IκBα binding pocket by establishing molecular binding with certain amino acids. Then, we assayed the systematic effects of PNC in an OVX mouse model. Micro-CT, TRAP and histological staining assays all showed that PNC significantly reduced bone loss in the OVX mice by suppressing osteoclastogenesis. Therefore, suppressive effects of PNC on osteoclasts was verified *in vitro* as well as *in vivo*.

Due to the “vicious cycle” in breast cancer bone metastasis (Roodman, 2004), we evaluated the effects of PNC on breast cancer cell-mediated osteoclastogenesis and on breast cancer cells. PNC inhibited osteoclast formation induced by human breast cancer MBA-MD-231 cells and that PNC could suppress the proliferation, invasion, migration of MBA-MD-231 cells and promote their apoptosis in a dose-dependent manner. Breast cancer cells can activate osteoclasts by producing RANKL or by stimulating osteoblasts to produce RANKL (Roodman, 2004; Ikeda and Takeshita, 2016; Ouyang et al., 2018). Our study showed that the inhibition of osteoclasts by drugs is due to suppression of the NF-κB pathway by PNCs, which is downstream of the RANKL pathway in osteoclast differentiation. Therefore, PNC might also inhibit osteoclast differentiation by inhibiting downstream signaling of RANKL secreted by breast cancer and osteoblasts. Consistent with a previous study, in the coculture system, we found that osteoclasts were fewer in number and smaller in volume than those of the osteoclast differentiation previously induced by RANKL (Figures 1B, 5D), which may be because our coculture system did not have osteoblasts, thus resulting in less osteoclastogenesis (Zhai et al., 2014).

From the above experimental findings and the theory of a “vicious cycle,” we hypothesized that PNC could prevent bone metastasis in murine tumors by suppressing osteoclast formation and function as well as the activity of MBA-MD-231 cells. Therefore, we established a bone metastatic model of breast cancer in nude mice to further verify whether PNC has a bone protective effect. Micro-CT scans established that, compared to the vehicle group, bone volumes of tumor-bearing mice in the PNC group were significantly higher. Furthermore, histological staining of the tumor-bearing mice showed that compared to the vehicle group, tumor growth was limited in bone marrow cavities and osteoclast formation near the binding site was suppressed in the PNC treatment group. Combined with the above experimental results *in vitro* as well as *in vivo* and the theory of a “vicious cycle,” we believe that PNC can inhibit bone metastases by inhibiting osteoclast formation, function as well as MBA-MD-231 cell proliferation, migration, and invasion and promoting their apoptosis. Previous drugs can inhibit osteoclasts to prevent bone metastases in breast cancer, while PNC may be more effective because it targets both osteoclasts and breast cancer cells (D’Oronzo et al., 2019; Nakai et al., 2019).

However, our investigation still has many limitations. First, an ideal breast cancer bone metastasis model should be able to reproduce all tumorigenesis as well as bone metastasis stages, but a mouse model of immunodeficiency might omit immune system regulation of the metastatic stage of breast cancer. Studies have used the clinically important 4T1-derived syngeneic mouse models of breast cancer bone metastases (Bidwell et al., 2012; Lee et al., 2014); however, there is a remarkable difference between the mouse and human microenvironments. In this paper, bone metastases were reproducibly generated in breast cancer bone metastasis animal models (Mundy 2001; Siclari et al., 2014), rarely occurred in other sites, which is valuable for our study. In addition, after PNC treatment of animal models, local histology revealed that there were no significant adverse effects or deaths in the PNC or other groups. However, the systemic side effects of PNC were not investigated. Therefore, in-depth studies involving more parameters of tissue specificity and biocompatibility are needed. Bone metastasis mechanisms are complex and are related to interactions among metastatic breast cancer cells, osteoblasts, and osteoclasts (Chen et al., 2010; Futakuchi et al., 2016). However, we have not yet studied whether PNC affects osteoblasts. Therefore, more studies should be conducted on PNC effects on osteoblasts in breast cancer bone metastasis. The formation and activation of osteoclasts has a relationship with increased reactive oxygen species (ROS) in osteoclasts (Ha et al., 2004; Lee et al., 2005; Yip et al., 2005). PNC has been found to act on the ROS pathway (Shen et al., 2021). However, the ROS pathway was not studied in our experiment. Therefore, we also need to further investigate the effect of PNC on ROS in osteoclasts. In addition, since we mainly found that drugs have effects on osteoclasts, we chose to study only the signaling pathway of osteoclasts and not the signaling pathways related to breast cancer cells, indicating the need for further studies to investigate PNC effects on breast cancer cell-associated signaling pathways. At present, the suppressive mechanisms of PNC on breast cancer cells have not been thoroughly explored. Some metastasis-associated genes are related to bone-homing (CXCR4), osteoclast recruitment (IL11), invasion (ADAMTS1 and MMP-1), and angiogenesis (CTGF and FGF5) (Casimiro et al., 2012). Abilities of metastasis-related genes to regulate metastasis have not been established, and more studies should investigate the effects of PNC on these genes.

Taken together, our work demonstrates for the first time that PNC can inhibit breast cancer cell invasion as well as migration and promote apoptosis *in vitro*, as well as attenuate osteoclast differentiation as well as function *in vitro* by blocking the NF-κB pathway. *In vivo*, PNC reduced breast cancer cell-mediated pathologic osteolysis in tumor-bearing mice, and decreased bone resorption in postmenopausal osteoporosis mice. Based on these findings, PNC may be a potential strategy for treatment of osteoporosis and breast cancer-associated osteolysis.

## Data Availability

The original contributions presented in the study are included in the article/supplementary material, further inquiries can be directed to the corresponding author.

## Abbreviations

BMD, bone mineral density; BMMs, bone marrow macrophages; BS/BV, bone surface/volume ratio; BV/TV, bone volume/tissue volume; CCK-8, cell counting kit-8; CT, computed tomography; DAPI, 4,6-diamidino-2-phenylindole; ERK, extracellular signal-regulated kinase; FBS, fetal bovine serum; JNK, c-Jun N-terminal kinase; MAPK, mitogen-activated protein kinase; M-CSF, macrophage colony-stimulating factor; NF-κB, nuclear factor-κB; OVX, ovariectomized; PBS, phosphate-buffered saline; PCR, polymerase chain reaction; PNC, Punicalin; RANKL, receptor activator of nuclear factor-κB ligand; ROS, reactive oxygen species; SD, standard deviation; Tb.N, trabecular number; Tb.Sp, trabecular space; Tb.Th, trabecular thickness; TBS, tris buffer solution; TRAP, tartrate-resistant acid phosphatase; V-ATPase-d2, ATPase H+ Transporting V0 Subunit D2.
