# Correction: Punicalin attenuates breast cancer-associated osteolysis by inhibiting the NF-κB signaling pathway of osteoclasts

**DOI:** 10.3389/fphar.2026.1678237

**Published:** 2026-01-19

**Authors:** Tao Li, Guangyao Jiang, Xuantao Hu, Daishui Yang, Tingting Tan, Zhi Gao, Zhuoyuan Chen, Cheng Xiang, Shizhen Li, Zhengxiao Ouyang, Xiaoning Guo

**Affiliations:** 1 Department of Orthopedics, The Second Xiangya Hospital, Central South University, Changsha, China; 2 Department of Immunology, Xiangya School of Medicine, Central South University, Changsha, China; 3 Department of Geriatrics, The Second Xiangya Hospital, Central South University, Changsha, China

**Keywords:** punicalin, osteoclast, osteoporosis, osteolysis, NF-κB, breast cancer, bone metastasis

There was a mistake in Figure 1B as published. Specifically, the images for the control and 31.25 μM in the BMMs group and the 125 μM splenocytes group were incorrectly selected during figure preparation. The affected images have been replaced with the correct representative images from the original experiments.

The corrected Figure 1 appears below.

**FIGURE 1:**
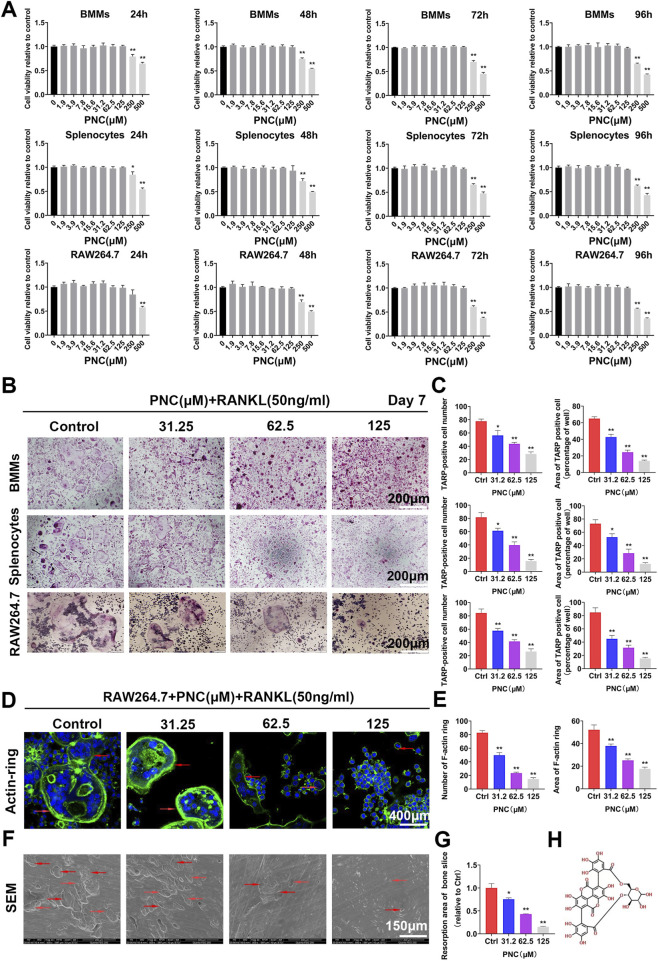
Nontoxic punicalin (PNC) inhibited RANKL-mediated osteoclast differentiation as well as function *in vitro*. **(A)** Cell viabilities of preosteoclasts after PNC treatments from 24 to 96 h (*n* = 3). **(B)** Osteoclastogenesis of three kinds of preosteoclasts, BMMs, splenocytes and RAW 264.7 cells *in vitro* after RANKL and PNC administration. **(C)** Quantification of osteoclast formation by PNC (*n* = 3). **(D)** F-actin ring formation after RANKL and PNC treatment. DAPI indicated cell nuclei (the red arrows indicate the F-actin ring). **(E)** Quantitative analysis of F-actin rings and osteoclast formation (*n* = 3). **(F)** Bone resorption pit formation after RANKL as well as PNC administration (the red arrows mark bone resorption pits). **(G)** Quantitative analyses of bone resorption pits (*n* = 3). **(H)** Structural formula of PNC. **p < 0.05, **p < 0.01 compared to controls.

The original article has been updated.

